# Complete de novo assembly of *Wolbachia* endosymbiont of Drosophila willistoni using long-read genome sequencing

**DOI:** 10.21203/rs.3.rs-4510571/v1

**Published:** 2024-06-14

**Authors:** Jodie Jacobs, Anne Nakamoto, Mira Mastoras, Hailey Loucks, Cade Mirchandani, Lily Karim, Gabriel Penunuri, Ciara Wanket, Shelbi L Russell

**Affiliations:** Department of Biomolecular Engineering, University of California Santa Cruz; Department of Biomolecular Engineering, University of California Santa Cruz; Department of Biomolecular Engineering, University of California Santa Cruz; Department of Biomolecular Engineering, University of California Santa Cruz; Department of Biomolecular Engineering, University of California Santa Cruz; Department of Biomolecular Engineering, University of California Santa Cruz; Department of Biomolecular Engineering, University of California Santa Cruz; Department of Ecology and Evolutionary Biology, University of California, Santa Cruz; Department of Biomolecular Engineering, University of California Santa Cruz

**Keywords:** *Wolbachia*, Drosophila, symbiosis, genomics

## Abstract

*Wolbachia* is an obligate intracellular α-proteobacterium which commonly infects arthropods and filarial nematodes. Different strains of *Wolbachia* are capable of a wide range of regulatory manipulations in many hosts and modulate host cellular differentiation to influence host reproduction. The genetic basis for the majority of these phenotypes is unknown. The *wWil* strain from the neotropical fruit fly, *Drosophila willistoni*, exhibits a remarkably high affinity for host germline-derived cells relative to the soma. This trait could be leveraged for understanding how *Wolbachia* influences the host germline and for controlling host populations in the field. To further the use of this strain in biological and biomedical research, we sequenced the genome of the *wWil* strain isolated from host cell culture cells. Here, we present the first high quality nanopore assembly of *wWil*, the *Wolbachia* endosymbiont of *D. willistoni*. Our assembly resulted in a circular genome of 1.27 Mb with a BUSCO completeness score of 99.7%. Consistent with other insect-associated *Wolbachia* strains, comparative genomic analysis revealed that wWil has a highly mosaic genome relative to the closely related wMel strain from *Drosophila melanogaster*.

## Introduction

*Wolbachia* is a gram-negative α-proteobacterium and is found as an endosymbiont in many arthropods and nematodes with a diverse range of effects on host phenotypes^1,2^. *Wolbachia* are maternally transmitted from host oocytes to the developing embryo^1^. *Wolbachia* strains manipulate host reproduction to promote their transmission to the next generation of hosts^2^. Subsequently, *Wolbachia* strains have strong affinities for host germline tissues^3^. Intriguingly, the

*Wolbachia* strain from the neotropical fruit fly *Drosophila willistoni*, *wWil*, selectively infects the host germline^4,5^. This unique tropism could be informative for understanding how *Wolbachia* localizes to and regulates the host germline with implications for vectorizing *Wolbachia* infections for biological control mechanisms.

The affinity of *w*Wil for host germ line cells is unique in comparison to closely related *Wolbachia* strains. Phylogenetic comparisons based on amplification of the wsp and ftsZ genes by PCR indicate that *w*Wil is closely related to the *w*Au strain found in *Drosophila simulans*^4^. However, unlike *w*Au, which infects both germline and somatic tissues in *D. simulans*, *w*Wil is exclusively found in the primordial germline cells of *D. willistoni* embryos^4^. Additionally, *w*Wil exhibits 100% maternal transmission in laboratory lines, which is attributed to *w*Wil’s tropism towards pole cells and selective infection of only the germ line^4^. Understanding the mechanism underlying the germ line specific tropism of *w*Wil could inform how other strains of *Wolbachia* localize to host tissue types.

This distinctive example of host cell specificity is crucial for understanding *Wolbachia*’s ability to colonize new hosts, with significant implications for biological pest control strategies. In *D. simulans* infected with non-native *Wolbachia* strains, the host genetic background has been shown to regulate the tissue tropism of the infection^5^. In native infections, *D. melanogaster* hosts *w*Mel *Wolbachia* infections in a broad range of cell types, infecting both somatic and germline tissues. Whereas in *D. willistoni*, *w*Wil demonstrates a restrictive infection pattern, targeting germline-derived cells^4,5^.

Despite the availability of numerous *Wolbachia* genomes, a complete *w*Wil genome is particularly important due to its unique germline-specific tropism. Here we present the first high-quality de novo assembly of *w*Wil obtained from nanopore sequencing of *w*Wil infected *in vitro D. melanogaster* cultures. In providing this genome, we seek to identify the genetic differences which exist between the *w*Wil and *w*Mel genomes and if those differences can provide insights into the mechanisms underlying *w*Wil’s germline-specific distribution.

## Results and discussion

### wWil genome assembly

We collected *Wolbachia w*Wil from *w*Wil-infected *Drosophila willistoni* embryos^6^ and introduced *w*Wil to immortalized *Drosophila melanogaster* JW18 cell culture cells with the shell vial technique^7^. We allowed the infection to stabilize by maintaining the culture for several weeks at 23°C, then collected the *w*Wil-infected cells from confluent cultures^7^. For each sample, 1.2 mL (at ~ 2e6 cells/mL) of cells were pelleted by centrifugation at 16,000xg for 10 minutes at 4°C. Following supernatant removal, DNA was extracted using the Wizard HMW DNA Extraction kit (Promega #A2920, Lot: 0000575812). Libraries were prepared with the Native Barcoding Kit V14 for Nanopore MinION R10 (Oxford Nanopore Technologies Cat #SQK-NBD114–24, Lot: NDP1424.10.0010) and sequenced on the Nanopore MinION Mk1B with a MinION R10 Version flow cell (FLO-MIN-114, Lot:11003064). We used Oxford Nanopore’s MinKNOW v23.07.8 software and with live basecalling with Guppy v7.0.8 (Fast model, read splitting ON) and a minimum read length of 200 bp and stopped sequencing after 36 hours. This resulted in 3.65 M reads with an estimated N50 of 1.11 kb and 2.6 Gb called with a minQ of 8.

Prior to genome assembly, we preprocessed the raw nanopore reads to remove host-derived sequences. Reads were aligned to the *D. melanogaster* reference genome (dmell-all-r6.46, retrieved from Flybase)^8^ with bwa mem^9^ v0.7.17. We used samtools^10^ v1.6 to sort and index the resultant file and remove reads which aligned to the host genome (samtools view -b -f 4). Preprocessed reads were output with bedtools^11^ v2.31.1 (bamtofastq). We removed sequencing duplicates with seqkit^12^ rmdup v2.7.0 and performed a de novo assembly of the *w*Wil genome with Flye^13^ v2.9 (preset, –nano-hq). We screened the assemblies for foreign genomic and adapter contamination using the NCBI Foreign Contamination Screen (FCS) toolkit version 0.5.0. We ran FCS-GX^14^ (taxa ID 953) and FCS-adaptor (run with --prok ag) which both found no evidence of contamination.

### Genome polishing and quality assessment

We generated Illumina short read whole genome sequence (WGS) data from JW18 cell culture cells stably infected with *w*Wil to polish the Nanopore assembly. Reads were aligned to the *w*Wil assembly and *D. melanogaster* reference^8^ (dmel6) simultaneously using *bwa mem* with default settings. Optical duplicates were marked with *sambamba*^15^. The reads aligning to dmel6 were discarded. The remaining reads were converted back to fastq format using samtools fastq, then re-aligned to the *w*Wil genome using minimap2 v2.26 with the settings -ax sr --cs --eqx. Reads with de (gap-compressed mismatch ratio) exceeding 0.04 were filtered out to remove mismapping and excess noise prior to polishing. The tool Pilon (v1.24) was run on these filtered alignments using default settings, producing the final polished assembly.

**Figure 1:**
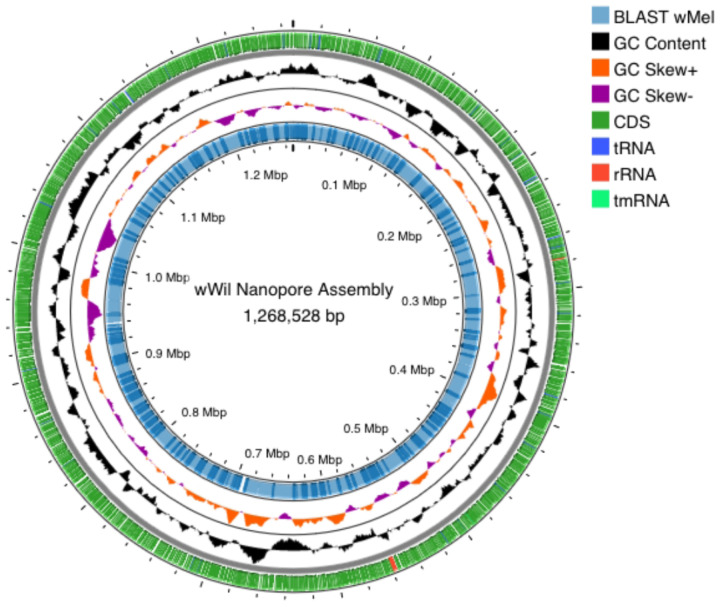
Map of the *Wolbachia w*Wil genome prepared using Proksee^18^. Circles in order from outer to inner show the following features: the position of coding sequences (CDS), open reading frames (ORF), tmRNA, tRNA, and rRNA genes (circle 1). GC content (circle 2) and GC skew plotted as the deviation from the average for the entire sequence (circle 3). The positions of BLAST hits detected through BLASTn comparisons of *w*Mel CP046925.1^22^ are shown in transparent blue, darker blue indicates regions which map to multiple regions in the *w*Mel genome (circle 4).

We assessed the quality of the polished assembly with BUSCO^16^ and annotated the genome with a standard work flow. BUSCO scores were calculated using the rickettsiales_odb10 database and v5.7.0. Polishing produced an improvement in BUSCO score from 98.6–99.7%. Default parameters were used for all software unless otherwise specified. We annotated the *w*Wil genome with Prokka^17^ v1.1.1 (kingdom:bacteria) to identify coding sequences (CDS), tRNAs, rRNAs, and tmRNA. GC Content and GC Skew were calculated with Proksee^18^ v1.1.2. We then aligned the *w*Wil genome against the *w*Mel reference genome (CP046925.1) with BLASTn with an expected value cut-off of 0.0001. We plotted these annotations with Proksee^18^ v1.1.2 to visualize the annotated genome (Fig. 1).

### Genome annotations and assessments

**Figure 2:**
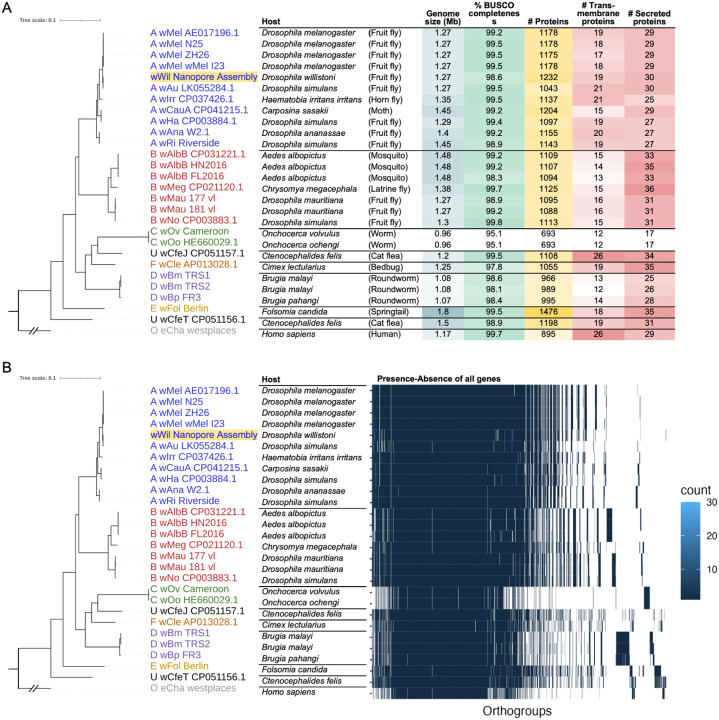
Comparative genomics of *w*Wil among other *Wolbachiaspecies*. (**a**) Phylogeny of *Wolbachia* genomes based on 470 single-copy orthologous genes (SCOs), with *w*Wil in supergroup A, along with genome metadata: host species and common name, genome size (Mb), BUSCO completeness score (%), total number of proteins, number of putative transmembrane proteins, and number of putative secreted proteins. (**b**) The same phylogeny as in A, with the presence-absence variation of all orthogroups shown. Whitespace indicates the absence of a gene in a particular *Wolbachia* genome.

**Figure 3:**
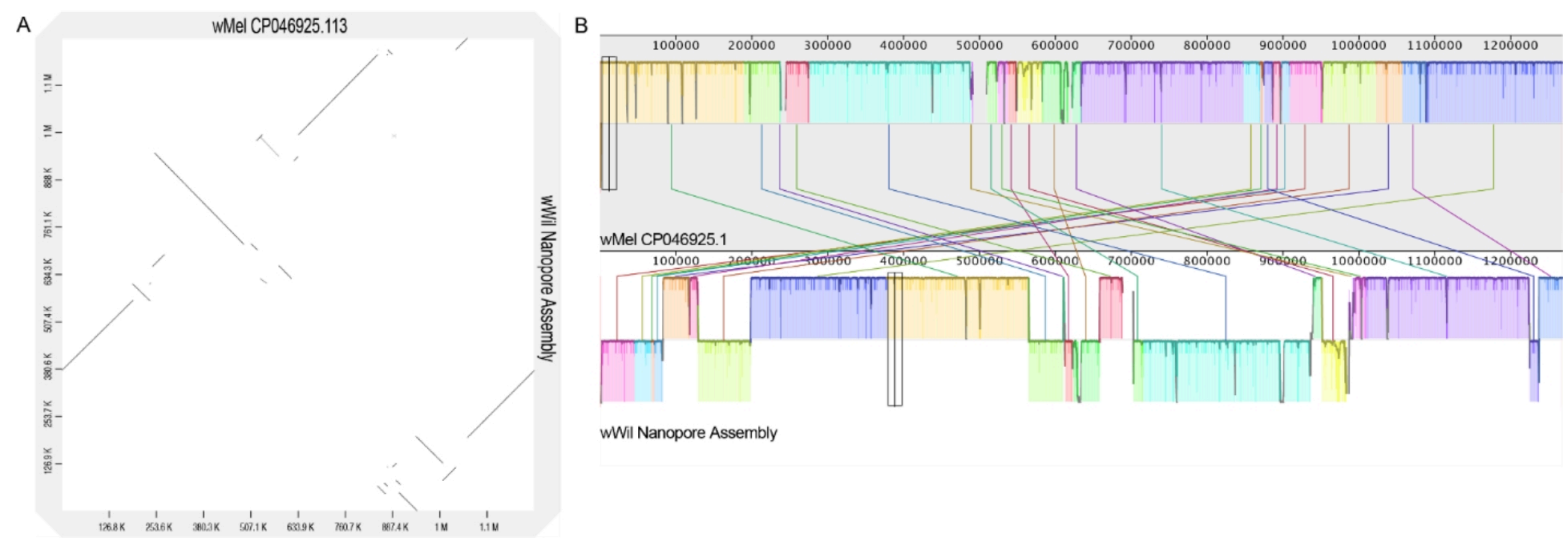
The comparison of *w*Mel CP046925.1^22^ with *w*Wil. (**a**) Dotplot generated with D-GENIES^28^ (**b**) Mauve alignment showing local collinear blocks (LCBs) identified along the circular genomes and joined with vertical lines.

To place our *w*Wil genome within the *Wolbachia* species phylogeny, we gathered a set of 27 circular, chromosome-level genome assemblies from many *Wolbachia* supergroups with broad host range^19^, and used *Ehrlichia chaffeensis* as an outgroup. Genes were annotated using the NCBI Prokaryotic Genome Annotation Pipeline^20^, and groups of orthologous genes (orthogroups) were identified across species with OrthoFinder2^21^. This produced a phylogeny based on single-copy orthologs, rooted on *E. chaffeensis*. Additionally, we utilized BUSCO^16^ analysis to characterize gene presence-absence variation across orthogroups. Our wWil assembly had a high BUSCO completeness score of 98.6% before polishing, which was comparable to the other circular, chromosome-level *Wolbachia* genomes. We found that the *w*Wil genome resides in *Wolbachia* supergroup A, alongside *w*Mel and many other fly-infecting species (Fig. 2A). Despite being closely related, alignment of the *w*Wil genome to the *w*Mel CP046925.1^22^ reference genome with Mauve^23^ (snapshot 2015-02-25.1) revealed many breaks in synteny between the genomes (Fig. 3). In general, our analysis showed a supergroup-specific pattern of gene presence-absence variation (Fig. 2B).

We also performed a brief assessment of putative secreted and membrane-bound proteins that could play a role in the *Wolbachia*-host interaction. Proteins with a signal peptide were identified by SignalP^24^, and proteins with a transmembrane domain were identified by TMHMM^25^. Those with a signal peptide and a transmembrane domain were classified as membrane-bound proteins, while those with a signal peptide but without a transmembrane domain were classified as secreted proteins. We then characterized presence-absence variation of putative secreted and membrane proteins within groups of orthologous genes across species. Finally, we identified variable sites in all proteins by calculating the Shannon entropy metric^26,27^, and compared the number of high-entropy sites in membrane and secreted proteins versus all proteins in general.

**Figure 4:**
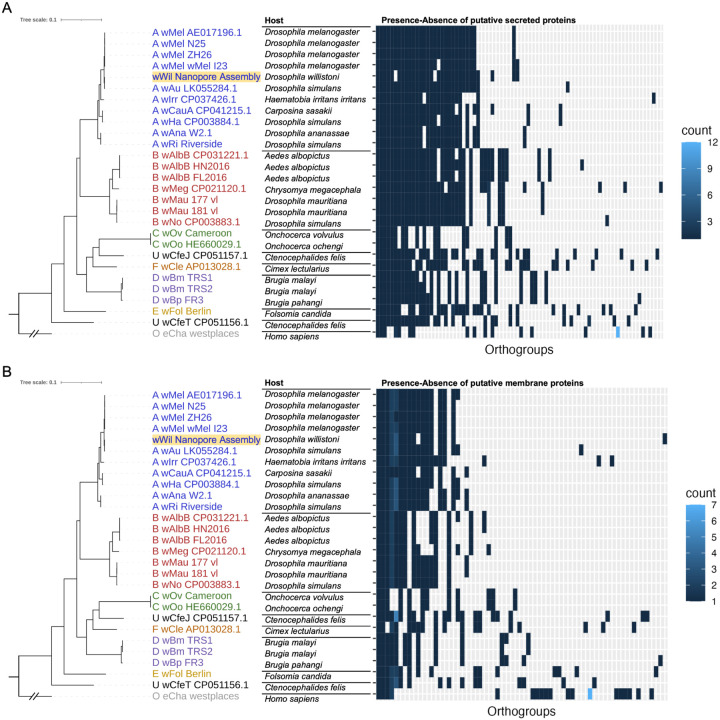
Presence-absence variation of putative (**a**) membrane protein and (**b**) secreted protein genes across orthogroups in *Wolbachia* species. As in Figure 2, the absence of a tile indicates the absence of a gene in a particular *Wolbachia* species.

**Figure 5:**
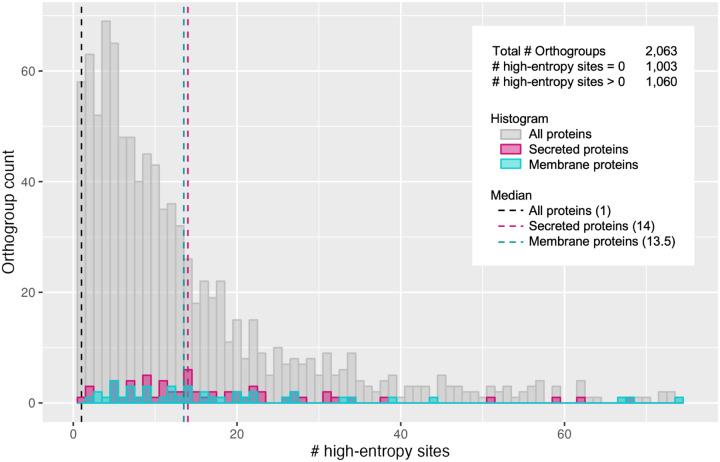
Variability of membrane proteins and secreted proteins compared to all proteins. Shown is a histogram of the distribution of orthogroups across the number of high-entropy (variable) sites in their protein sequence alignment. Orthogroup counts are plotted separately for all proteins (gray), secreted proteins (pink), and membrane proteins (blue), with median number of variable sites represented by dashed lines of the respective colors. There were 1,003 orthogroups that did not contain any variable sites, which are not included in the plot.

Just as for all genes, there was a supergroup-specific pattern in presence-absence variation for both membrane-bound and secreted proteins across *Wolbachia* species (Fig. 4). Additionally, membrane and secreted protein groups had many variable sites compared to all proteins in general. The median number of variable sites in an orthogroup across all *Wolbachia* genes was one, while the medians for secreted and membrane proteins were 14 and 13.5 variable sites respectively (Fig. 5). This analysis revealed proteins with many sites that vary across diverse *Wolbachia* species with a wide host range, and thus provides candidates for further interrogating *Wolbachia*-host interactions at the molecular level.

## Data Availability

The assembled genome and the raw long and short reads are available in BioProject PRJNA1107195.
